# Investigating the impact of the quantity of wet and dry cycles on the mechanical characteristics and fracture variations of sandstones

**DOI:** 10.1038/s41598-024-63577-9

**Published:** 2024-06-10

**Authors:** Ruiyu He, Xin Tang, Hong Yin, Yujia Qin, Zhengchao Guo, Li Fang, Xiaoyi Zhou, Yuerong Zhou

**Affiliations:** 1 School of Civil Engineering, Chongqing Three Gorges University, Chongqing, 404199 China; 2 Wanzhou District Meteorological Bureau, Chongqing, 401147 China

**Keywords:** Sandstone, Uniaxial compression, Dry–wet cycle, Mechanical characteristics, Crack changes, Numerical simulation, Engineering, Civil engineering

## Abstract

The sandstone is in a state of dry–wet cycle under the repeated action of rainfall, and its mechanical properties are deteriorated to varying degrees, which causes cracks in the sandstone. Therefore, it is of great significance to study the mechanical properties and fracture propagation of sandstone under the action of dry–wet cycles. Currently, there are limited studies using numerical simulation methods to study the fracture extension of rocks under various dry and wet cycling conditions.Therefore, in this paper, the effects of different amounts of dry and wet cycling on the mechanical properties and fracture behavior of sandstone are investigated through uniaxial compression tests and numerical simulations of fracture extension. The findings indicate that the deformation stage of sandstone remains unchanged by the dry–wet cycle. The uniaxial compressive potency and coefficient of restitution gradually diminish as the quantity of cycles rises, while the Poisson's ratio exhibits the opposite trend, and the impact on the mechanical performance of sandstone wanes with cycle increments, and the correlation coefficient surpasses 0.93, signifying a substantial influence of the dry–wet cycle on sandstone's mechanical performances. The discrepancy between the numerical simulation and experimental results is minimal, with a maximum error of only 3.1%, demonstrating the congruence of the simulation and experimental outcomes.The mesoscopic examination of the simulations indicates that the quantity of fractures in the sandstone specimens rises with the escalation of dry–wet cycles, and the steps of analysis linked to crack inception and fracture propagation are accelerated, and the analysis steps from fracture initiation to penetration are also reduced.

## Introduction

Sandstone is mainly formed by the deposition of clastic sediments under the influence of pressure and temperature, and it is commonly encountered in the construction of tunnels and mining projects in China. Due to factors such as environmental pollution, frequent rainfall, the presence of groundwater, and high temperatures, the rock and soil mass is subjected to long-term damage from alternating wet and dry conditions, which adversely affects its safety and stability^1,2^. The long-standing effects of dry–wet cycle accelerate the destruction of the internal structure of the geotechnical body, constantly impacting the safety of engineering implementation^3^. Therefore, it is important to study the damage deterioration of geotechnical bodies under wet and dry cycling.

Currently, many scholars have investigated the effects of wet and dry cycling on the damage deterioration of geotechnical bodies through indoor experiments. Chen et al.^4^ experimentally investigated the strength deterioration of red sandstone under different dry and wet cycles. Gao et al.^5^ have investigated the factors of damaging deformation of sandstone under wet and dry cycling through experiments. Li et al.^6^ revealed the static properties of red shale through uniaxial compression experiments. Hu et al.^7^ experimentally investigated the pore structure development and uniaxial compressive mechanical properties under different number of freeze-thaw cycles. Zhang et al.^8^ revealed the uniaxial conventional mechanical properties of sandstone under wet and dry cycling conditions through experiments. Cheng et al.^9^ established a theoretical model for evaluating the deterioration of the mechanical properties through experiments. Ren et al.^10^ investigated the destruction mechanism of rocks through triaxial compression tests. Ma et al.^11^ investigated the asymptotic deformation characteristics of rocks during different quantities of wet and dry cycling. Luo et al.^12^ investigated the mechanical characteristics of black shale with different basal surfaces by using indoor triaxial compression tests. Wen et al.^13^ revealed the damage evolution and damage mechanisms of rocks by using tests. Wei et al.^14^ studied the mechanical properties of carbonaceous shale under wet and dry cycling conditions by using tests. Li et al.^15^ investigated the energy evolution and three-dimensional strength damage criterion of shale through experiments. Liu et al.^16^ studied the energy fluctuation of rock during wet and dry cycling through experiments.

Due to the complexity of practical problems, field tests are sometimes difficult to carry out, so many scholars choose numerical simulation to study the damage deterioration of rocks. Dagdelenler et al.^17^ used numerical simulation to establish a nonlinear model for predicting the degree of weathering of granite by the use of mechanical parameters. Miao et al.^18^ used numerical simulation to establish a structural model of rock damage under uniaxial compression and erosion by chemical solutions. Cai et al.^19^ used numerical simulation to simulate the peak strength and participation strength of jointed rocks and quantitatively analyzed the damage caused by microseismicity in underground engineering excavation. Due to the complexity of the damage degradation mechanism of rocks, the damage of geotechnical bodies in actual engineering is often the process of the emergence, expansion, confluence and penetration of internal cracks. Therefore, it is of great significance to study the crack extension of rocks under dry and wet cycles. The actual process of crack extension in rocks under wet and dry cycling conditions is usually difficult to observe and measure directly, but numerical simulation can help researchers to explore the different stages of crack extension by adjusting the model parameters and conditions. Zhou et al.^20^ and Zhang et al.^21,22^ have investigated the damage mechanism and crack extension of brittle rocks by numerical simulation. Carter et al.^23^ used the USR coefficient of rock strength to investigate the spreading data of different cracks around the hole. Wong et al.^24^ systematically analyzed the effects of pre-set crack length, inclination, and sample width on crack initiation, extension, and damage modes in fractured rocks through numerical simulations. Zhou et al.^25^ investigated the process and characteristics of crack initiation, extension and penetration in rocks. Guo et al.^26^ explored the effects of wet and dry cycles on the stability of rocks around tunnels using numerical simulation. Fu et al.^27^ investigated the crack extension and mechanical response of marble under three different cyclic loads by numerical simulation. Huang et al.^28^ investigated the changes of physical properties of sandstone under acidic wet/dry cycling and discrete element simulation. We found that the main shortcoming in the current research is a certain lack of numerical simulation of crack extension in rocks under dry and wet cycles. Most of the existing studies focus on simulations in the case of acidic solutions, cavity rocks and fissured rocks, while numerical simulations of crack extension in intact rocks under wet and dry cycling have not been adequately investigated.

The innovation of this study is that the effects of different amounts of wet and dry cycles on rock mechanical properties and crack extension were investigated by uniaxial compression tests and numerical simulations. Through this novel research method, we are able to gain a deeper understanding of the mechanisms and laws of crack extension in intact rocks under wet and dry cycling conditions. This will provide a more comprehensive and accurate reference for the field of rock engineering, which will help guide the development of related engineering practices and optimized design. Therefore, this study fills the gap in current research and makes an important contribution to the development of the field.

### Overview of the region

**Figure 1:**
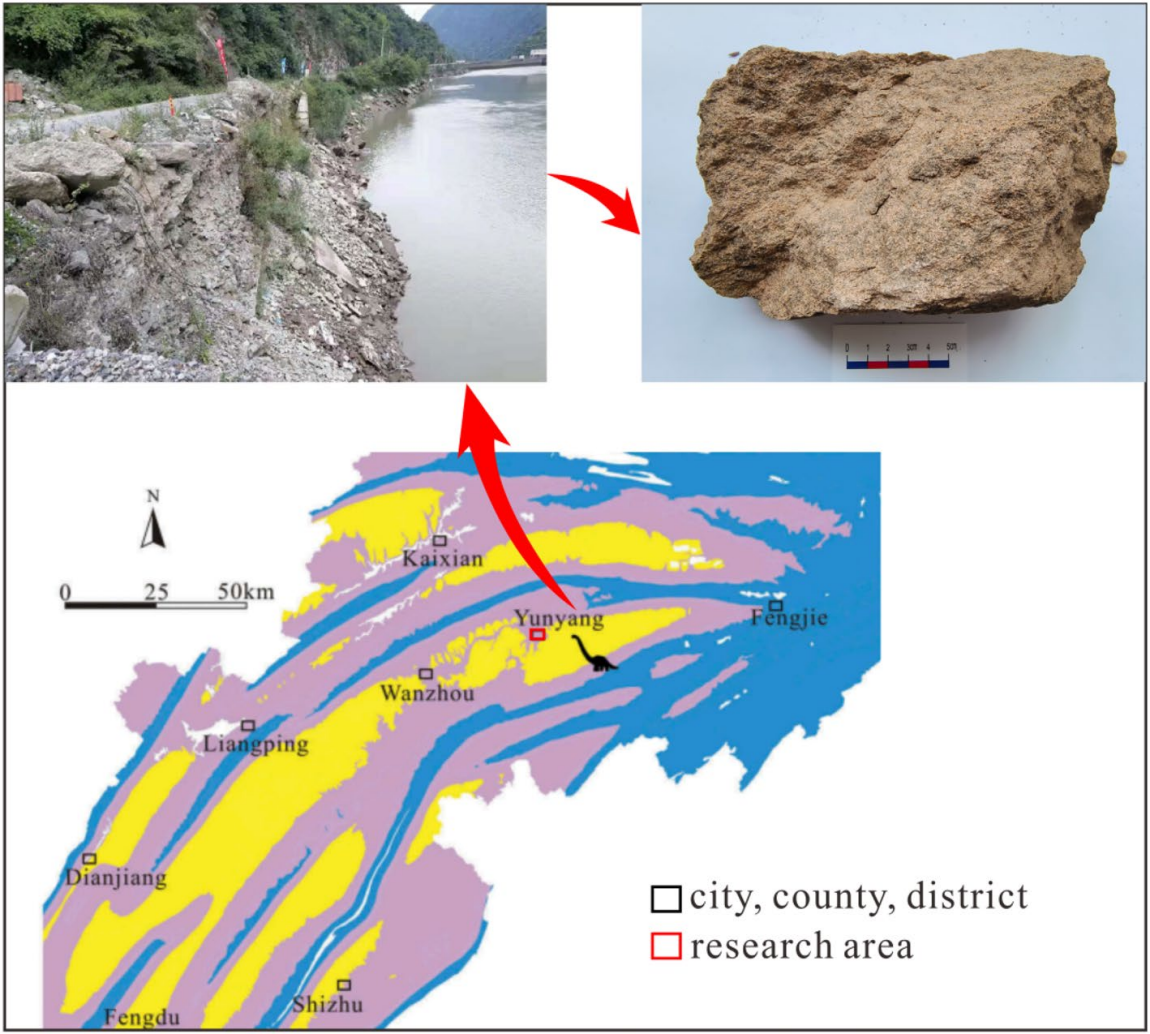
Location of the study area and samples.

The study area is situated in Yunyang County, within the central area of the Three Gorges Reservoir Region, to the northeast of Chongqing. The samples were obtained from the weathered sandstone of the Suining Formation within the study area, which exhibits a yellowish-gray color (see Fig. 1).

## Experimental methods

### Sample preparation

**Figure 2:**
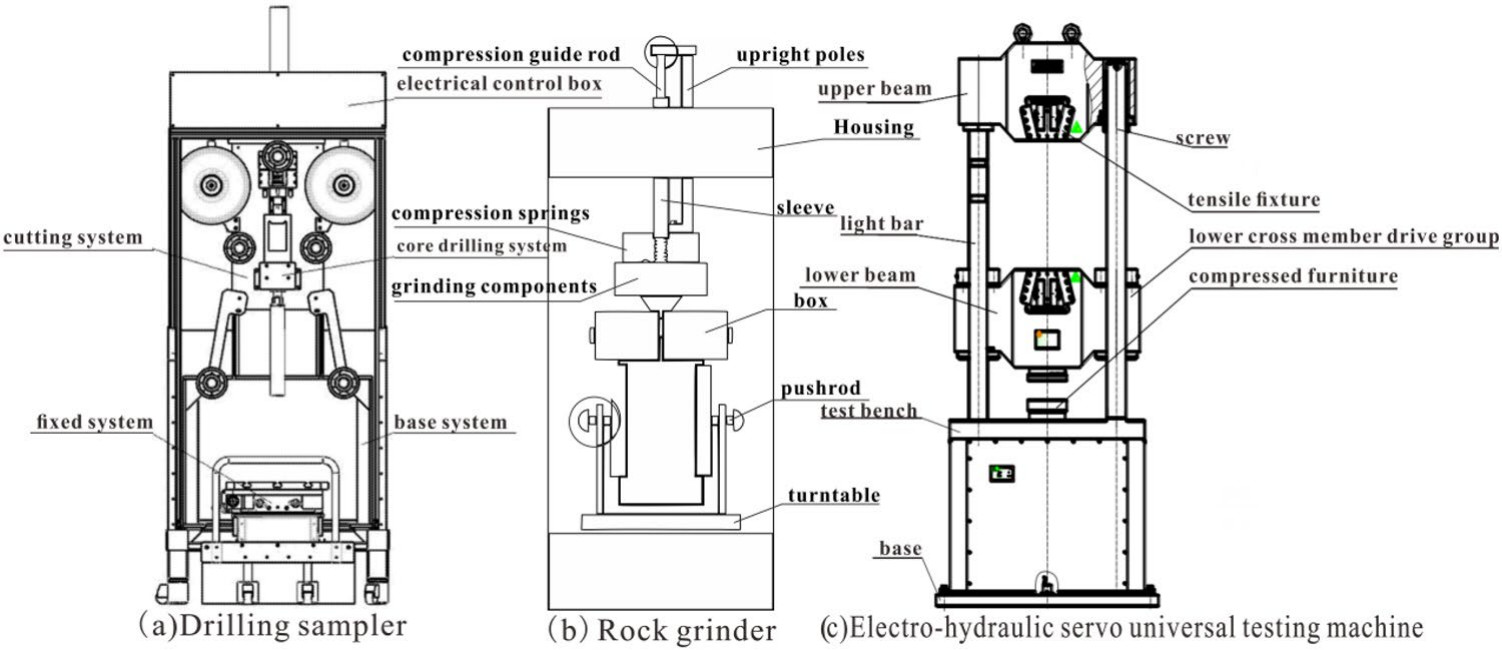
Schematic diagram of the experimental equipment: (**a**) drilling sampler, (**b**) rock grinder, (**c**) electro-hydraulic servo universal testing machines.

The rock samples were processed using a drilling sampling machine, which consists of a base system, a drill core system, an electrical control box, a cutting system, and a fixing system, as depicted in Fig. 2a. The specimen is a conventional cylinder with a 50 mm diameter (D) and 100 mm height (h), in compliance with the specifications outlined in international rock mechanics testing standards. Subsequently, the rock samples were polished using a grinding machine, which consists of equipment uprights, casings, sleeves, boxes, push rods, turntables, grinding components, compression springs, and compression guide rods, as depicted in Fig. 2b. The flatness of the polished end surface after grinding was required to be ≤ 0.02 mm, with a diameter error accuracy of ≤ 0.1 mm.

### Wet-dry cycle testing

Considering the complex physicochemical reactions resulting from the interaction between water and rock, natural waters such as groundwater, mineral water, river water, and rainwater can affect the experiments. Therefore, purified water was utilized in this investigation to perform examinations on the rock specimens. The wet-dry cycle was set at 0 cycles (dry), 1 cycle, 3 cycles, 5 cycles, and 10 cycles.The sample treatment for the specific test procedure was as follows:

1. No wet-dry cycle: The samples without wet-dry cycle are used as the control group, the temperature of the control group is set at 20 ℃, the humidity is set at 50%;
2. dry and wet cycle processing: the sample is placed in a vacuum drying oven, set the temperature of the drying oven is 105 ℃, set the drying time of 24 h; remove the sample to the temperature of 20 ℃ and humidity 50%, and then put into the vacuum saturated device, set the temperature of 35 ℃, humidity 80%, set the time of immersion for 24 h, and then the device automatically discharged water until the sample reaches the dry state. This process of soaking followed by drying to complete dryness represents a single iteration of the wet/dry cycle check. The iteration is repeated according to the number of wet/dry cycles specified, e.g. 1, 3, 5, 10.

### Uniaxial compression testing

The uniaxial compression test was carried out on the samples treated with wet and dry cycles for 0, 1, 3, 5 and 10 times, respectively, to record the mechanical parameters of the rocks at different times, and the samples treated with wet and dry cycles for 0 times were used as the control group, using the method of controlling variables, the variable in the experiment was the number of wet and dry cycles, and the effect of wet and dry cycles on the mechanical properties of the rocks was comparatively analyzed.The uniaxial compression tests using electro-hydraulic servo universal testing machines, which consists of an upper crossbeam, light rod, lower crossbeam, test platform, base, screw, tension fixture, lower crossbeam transmission group, and compression fixture, as shown in Fig. 2c. The testing apparatus can deliver an axial load spectrum of 0–1500 kN, a confining pressure range of 0–60 MPa, and a permeating pressure range of 0–60 MPa. The axial load control method was used during the test, with a loading rate of 6.12 bar/min.

### Numerical simulation

**Figure 3:**
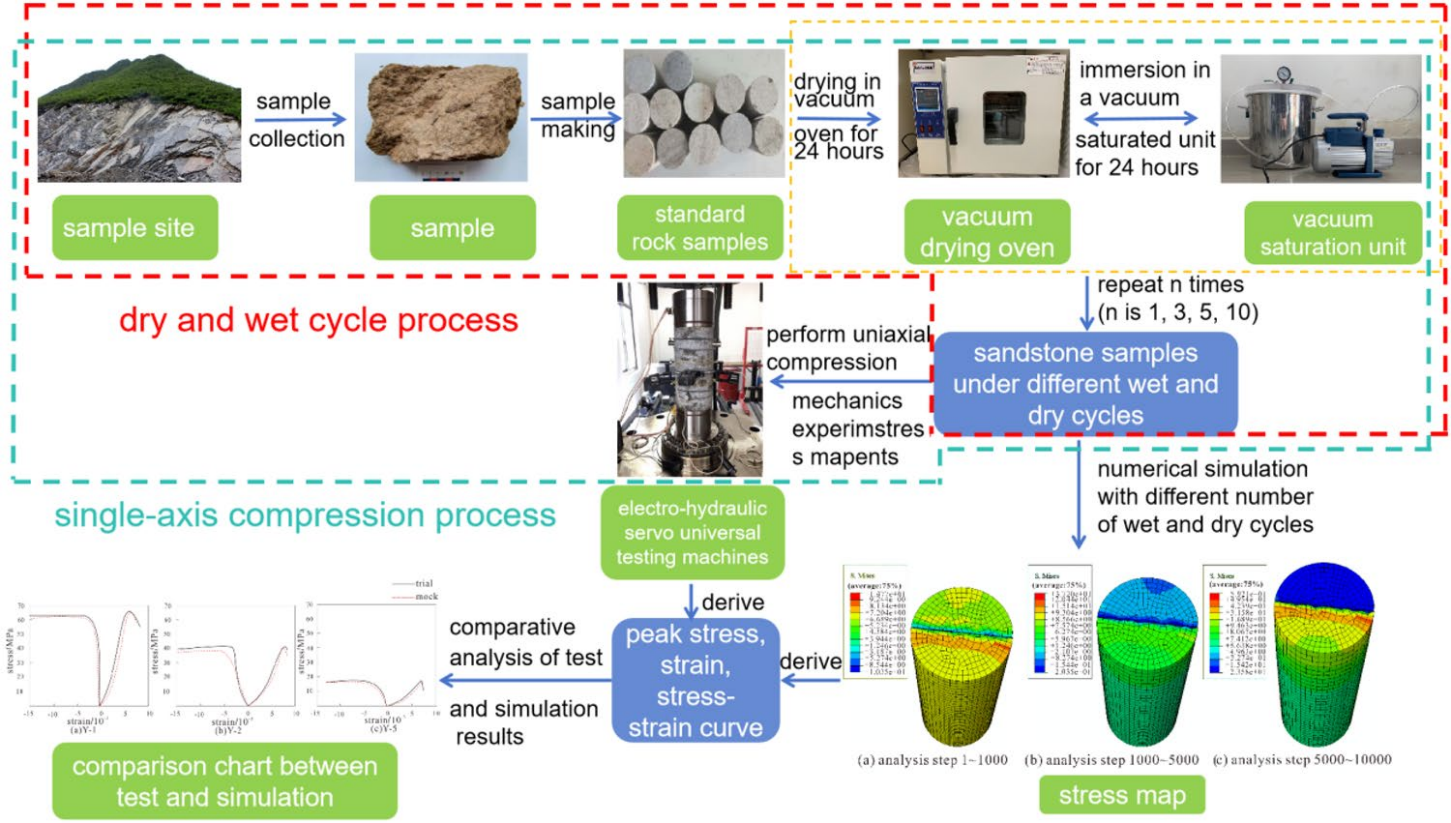
Flowchart of experiments and numerical simulations.

Because of the limited experimental conditions, it is hard to observe the deformation and cracking change process of rocks under uniaxial compression test. Therefore, The finite element simulation tool ABAQUS was employed to model the deformation and failure progression of rocks following 1 and 10 wet-dry cycles, as well as untreated rocks. Combined with the above, we finally drew the integrated flow chart of test and numerical simulation (Fig. 3), which includes the flow charts of wet and dry cycles and uniaxial compression test as well as the flow chart of numerical simulation.

**Figure 4:**
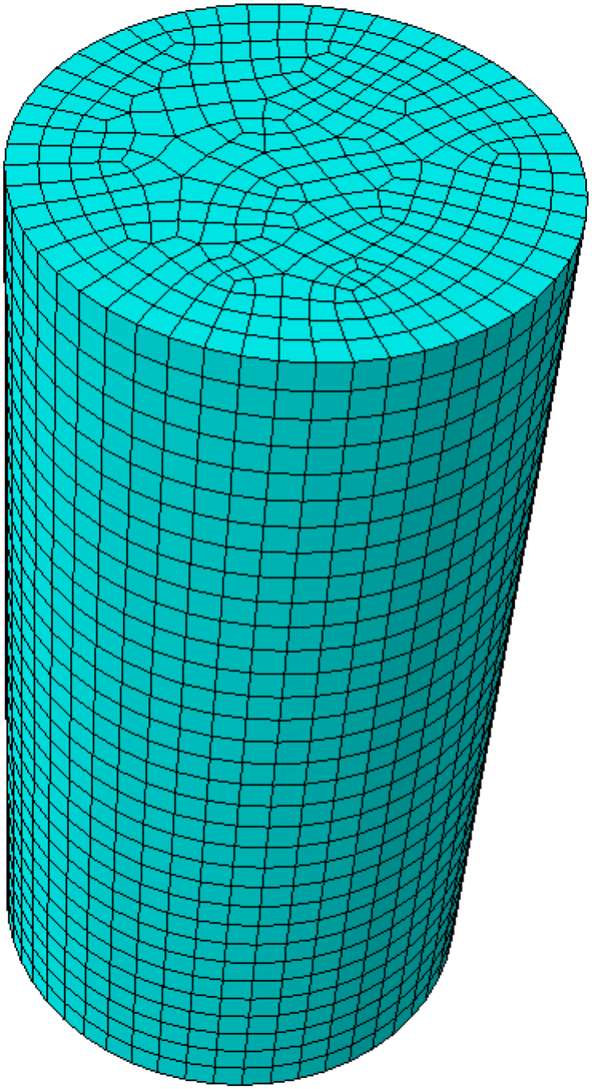
Numerical calculation of the finite element model.

The main steps of numerical simulation using Drucker-Prager constitutive relation are as follows:

1. Create a part: create a 3D cylinder with a diameter of 50 mm and a height of 100 mm (as shown in Fig. 4).
2. Define material properties: create material property 1, define parameters such as elastic modulus, compressive strength and Poisson’s ratio of rock materials (as shown in Table 1), create material property 2, and define the density of water as 1 g/cm^3^.
3. Assembling and setting boundary conditions: setting assembly, the boundary conditions are fixed at the bottom, the upper part applies vertical downward pressure, and the amplitude function is set.
4. Analysis step setup: Set analysis step 1 as a static analysis step at 105 °C for 24 hours to simulate dry loading, and set analysis step 2 as a dynamic analysis step at 35 °C and 80% humidity for 24 hours to simulate immersion.
5. Alternate setting of loading steps: In the loading module, at the end of each loading step, the starting conditions of the next loading step are defined by setting the output option to achieve alternating drying and soaking states.
6. Meshing: Set the grid to C3D8R, a total of 10,065 grid elements.
7. Define the number of cycles: In the job module, select your job and edit, select the Steps tab, and then select the loading step you want to set the cycle, in the settings of the loading step, you can find the Number of cycles option, control the number of dry and wet cycles by setting the number of cycles, and set the judgment condition of the cycle between each drying and soaking loading step in the “Field Output Request” of the output tab, whether to end the cycle.
8. Output profile: In the visualization module, the stress profile is output.

**Table 1:** Numerical simulation model parameters.

Various amounts of dry–wet cycles	Sample number	Uniaxial compressive potency σc/MPa	Coefficient of restitution E/GPa E/GPa	Poisson’s ratio µ
0	Y-1	60.53	10.81	0.20
1	Y-2	42.35	7.40	0.27
10	Y-5	23.54	3.30	0.19

### Inspection results

#### Stress–strain curve

According to the uniaxial compression test, the specific mechanical parameters under different drying-wetting cycles were obtained (Table 2).

**Table 2:** Uniaxial compression test results.

Various amounts of dry–wet cycles	Sample number	Uniaxial compressive potency σ_c_/Mpa		Coefficient of restitution E/GPa E(GPa)		Poisson’s ratio µ	
		Single value	Mean	Single value	Mean	Single value	Mean
0	Y-1	57.94	60.53	11.32	10.81	0.19	0.20
		60.73		10.95		0.20	
		62.39		10.16		0.22	
1	Y-2	40.83	42.35	7.99	7.4	0.24	0.24
		42.56		7.39		0.23	
		43.65		7.08		0.25	
3	Y-3	32.94	33.35	5.64	5.34	0.24	0.25
		33.13		5.34		0.26	
		33.98		5.03		0.25	
5	Y-4	26.84	27.87	4.17	4.06	0.25	0.26
		28.03		4.03		0.26	
		28.75		3.98		0.27	
10	Y-5	22.45	23.54	3.53	3.30	0.27	0.27
		24.34		3.38		0.26	
		23.82		2.98		0.28	

**Figure 5:**
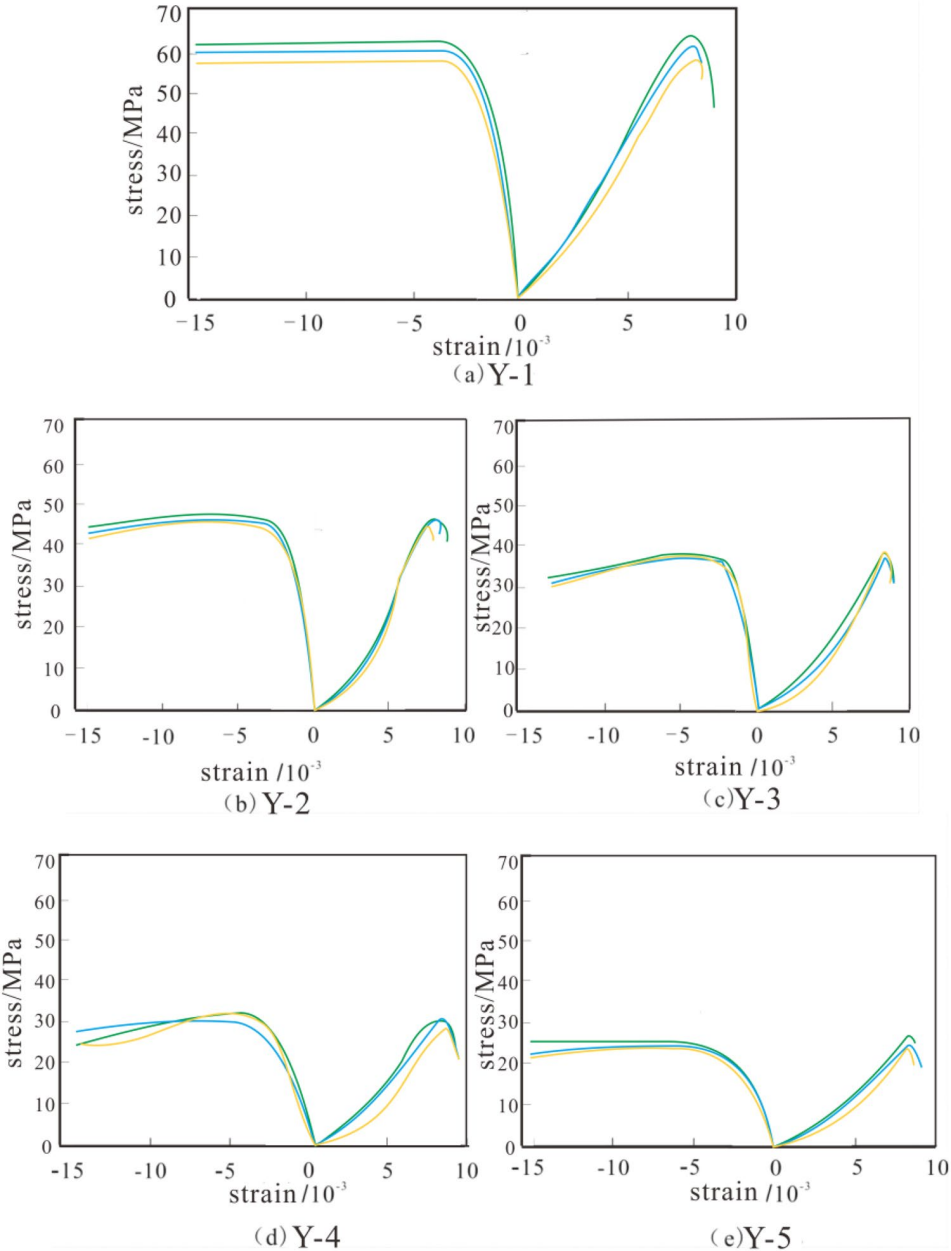
Stress-deformation diagrams of sandstone under varying quantities of dry–wet cycles: (**a**) Y-1, (**b**) Y-2, (**c**) Y-3, (d) Y-4, (**e**) Y-5.

**Figure 6:**
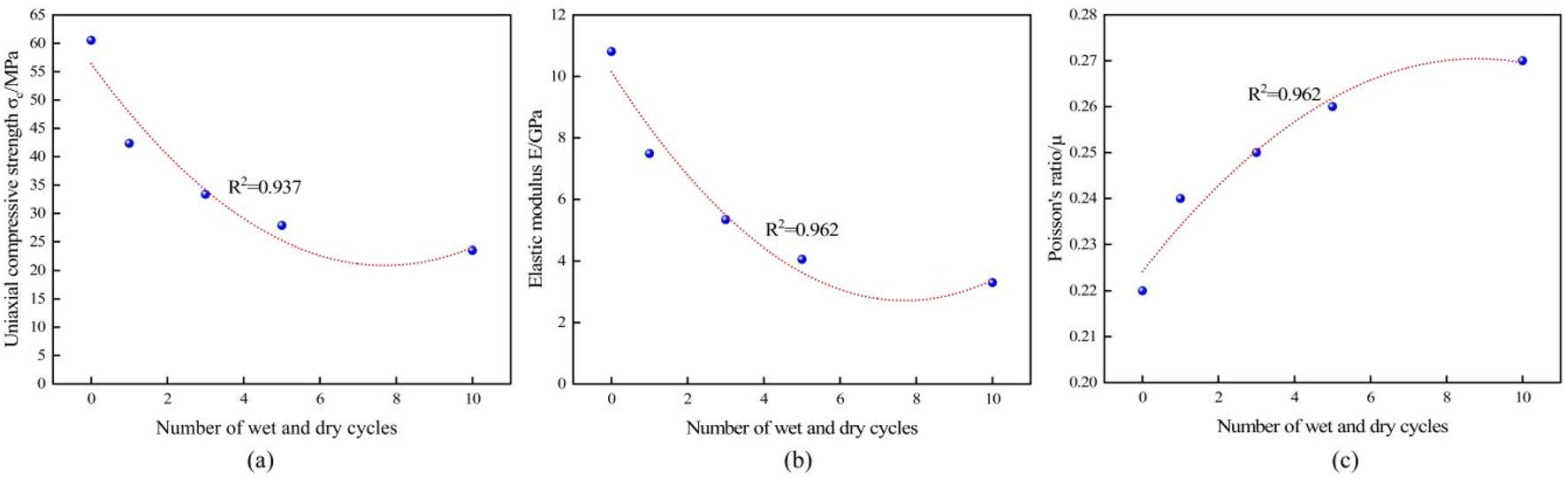
The mechanical properties index and the number of cycles are fitted to the curve: (**a**) uniaxial compressive potency, (**b**) coefficient of restitution, (**c**) Poisson’s ratio.

Based on the aforementioned test outcomes (Table 2), the stress–strain diagrams illustrating the entire unidirectional compression process of sandstone samples under varying wet-dry cycles, along with the correlation curves illustrating the correlation between the quantity of wet-dry cycle and the Poisson's ratio (μ) of uniaxial compressive potency (σ_c_), coefficient of restitution (E), and Poisson's ratio can be acquired, as displayed in Figs. 5, 6a–c.

From Fig. 5, it is evident that the stress–strain profiles of the sandstone samples, irrespective of whether they undergo dry–wet cycle, follow the typical pattern of rock uniaxial compression tests, experiencing five stages: initial compaction, linear elastic deformation, stable crack expansion, pre-peak plastic hardening, and post-peak failure. Firstly, in the initial stage of compaction, as the rock belongs to discontinuous material with poor homogeneity, there are many defective regions in the structure, such as cracks, fissures and holes. When the rock sample is subjected to external loading, the internal defective regions are gradually closed, and the rock sample is gradually compressed and compacted; followed by the linear elastic deformation stage, in which the internal defective regions of the rock will be established on the basis of the previous compaction stage, and the stress-strain relationship is approximately proportional under continuous compaction, i.e., the curve is basically a straight line segment, and the stress-strain relationship can be used as a linear function to estimate; then the stable crack extension stage, with the increase of external load, new cracks and fissures begin to appear internally and continue to develop, expand and penetrate; followed by the pre-peak plastic deformation hardening stage, where the rock continues to deform plastically but has not yet reached its peak strength; and finally, the post-peak deformation stage, where the rock rapidly decreases from its peak strength, leading to specimen destruction. The results of this experiment are consistent with the findings of numerous scholars^29–33^, demonstrating that the findings are reasonable and plausible.

Comparing the stress-strain curves of sandstone specimens under different cycle times, it is found that the peak stress of sandstone specimens gradually decreases with the increase of cycle times, which may be due to the fact that microcracks and pores in the sandstone specimens will gradually expand under the action of multiple wet and dry cycles, resulting in changes in the stress distribution inside the rock, which leads to a gradual decrease in the peak stress of the rock. In addition, the wet and dry cycles will also cause the friction and interaction force between the particles in the rock to increase, thus reducing the cohesion and compressive strength of the rock, which is also one of the reasons for the reduction of the peak stress. By observing and analyzing the results of three tests with the same experimental setup in Fig. 5, it can be found that the data of the three tests are very similar, and the change rule of the stress-strain curve is also the same, which indicates the reliability and consistency of the experimental data.

 On this basis, the mechanical behavior of rocks under the action of dry and wet cycles can be studied in depth to further understand the mechanism. These research results can be applied to practical engineering problems, for example, in the field of geotechnical engineering, according to the mechanical parameters of rocks under different dry and wet cycling conditions, suitable geotechnical engineering structures can be designed and their behaviors can be predicted in practical engineering environments, so as to better ensure the safety and stability of the project.

### Mechanical performance indicators

After analyzing Table 2, Fig. 6a and b, the uniaxial compressive strength of the rock samples decreased from the initial value of 60.53–42.35 MPa, or 30.03%; the coefficient of recovery of the rock decreased from the initial value of 10.81–7.49 GPa, or 30.71%; and the Poisson's ratio increased from the initial value of 0.20–0.24. After three dry–wet cycles, the uniaxial compressive strength of the rock samples decreased from 42.35 to 33.35 MPa, or 21.25%. After three wet and dry cycles, the uniaxial compressive strength of the rock samples decreased from 42.35 to 33.35 MPa, with a decrease of 21.25%; the coefficient of recovery decreased from 7.49 to 5.34 GPa, with a decrease of 28.70%; the Poisson’s ratio increased from 0.24 to 0.25. After five wet and dry cycles, the uniaxial compressive strength of the rock samples decreased from 33.35 to 27.87 MPa. After five dry and wet cycles, the uniaxial compressive strength of the rock samples decreased from 33.35 to 27.87 MPa, with a decrease of 16.43%; the coefficient of restitution decreased from 5.34 to 4.06 GPa, with a decrease of 23.97%; the Poisson’s ratio increased from 0.25 to 0.26 accordingly; and the uniaxial compressive strength and modulus of elasticity of the rock samples decreased from 15.54 to 23.45%, with a decrease of 23.54% to 3.30 GPa, with a decrease of 23.45%. This may be due to the increase of cracks and holes within the sandstone caused by wet and dry cycling, resulting in irreversible damage, and the damage increases with the increase in the number of cycles, making the sandstone resistance to deformation weakened, which results in the uniaxial compressive strength and modulus of elasticity of sandstone decreases with the increase in the number of wet and dry cycles.

It was found that the decrease in the number of wet and dry cycles was associated with the decrease in the uniaxial compressive strength and coefficient of recovery of the rock samples, and this effect decreased with the increase in the number of cycles. Linear regression analysis of the number of wet and dry cycles with uniaxial compressive strength and modulus of elasticity were fitted with 0.937 and 0.962, respectively, indicating that the number of wet and dry cycles has a great influence on the mechanical properties of rocks. Through comparative analysis, it was found that the results of this study are consistent with the results of previous studies^31–37^. This may be because there are many microcracks inside the sandstone, and after dry–wet cycles, water enters the cracks, softening the internal particles of the sandstone through water–rock interaction, thereby reducing its mechanical performance indicators.

Figure 6c shows that the Poisson’s ratio of the sandstone increases with the number of wet and dry alternations, and the effect of this trend decreases with the number of cycles. The correlation coefficient obtained from a linear regression fit was 0.962, indicating a good fit. This indicates that wet and dry cycling has a significant effect on the Poisson’s ratio of the sandstone. This is consistent with the results of related scholars.^35–37^.

### Stress cloud diagram

The process of deformation and destruction of rocks involves the creation, extension, joining, penetration and sliding of internal microcracks^38,39^. For indoor uniaxial compression tests of rocks, as the rock undergoes loading, numerous microcracks develop within its interior. With the increase in axial stress, these cracks progressively propagate until the rock ultimately fractures.

**Figure 7:**
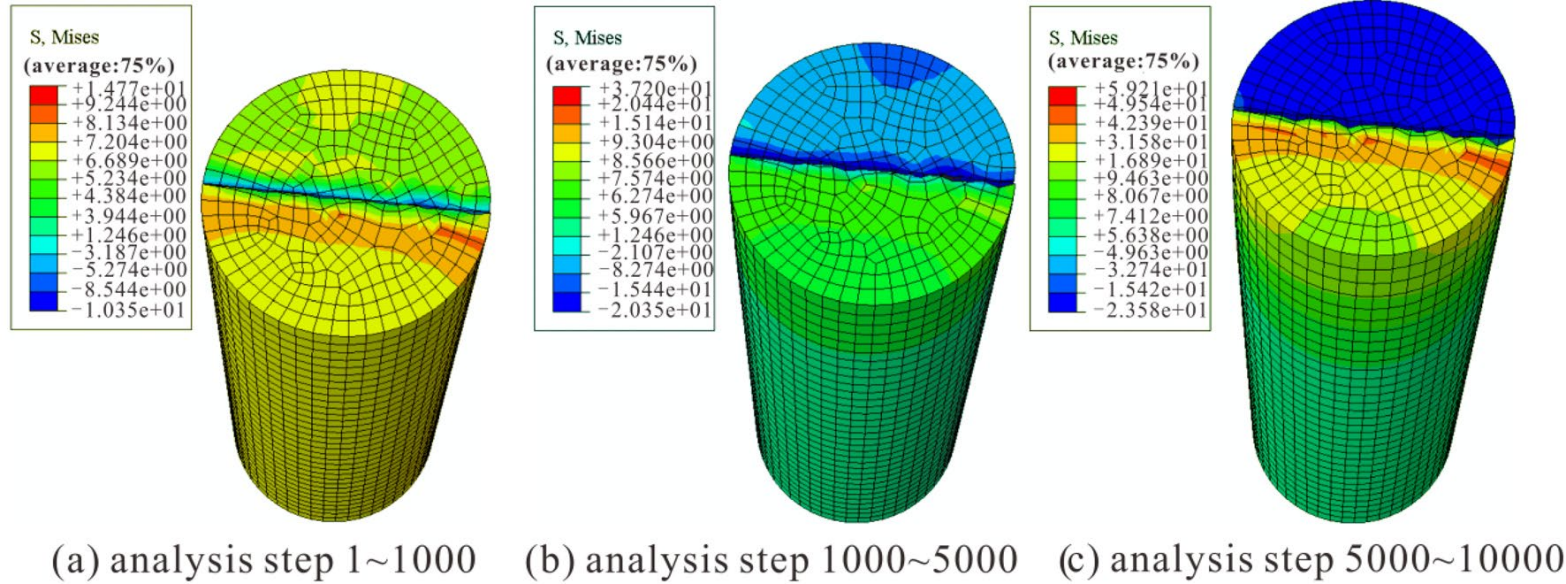
Numerical simulation stress cloud diagram without dry–wet cycle effect.

**Figure 8:**
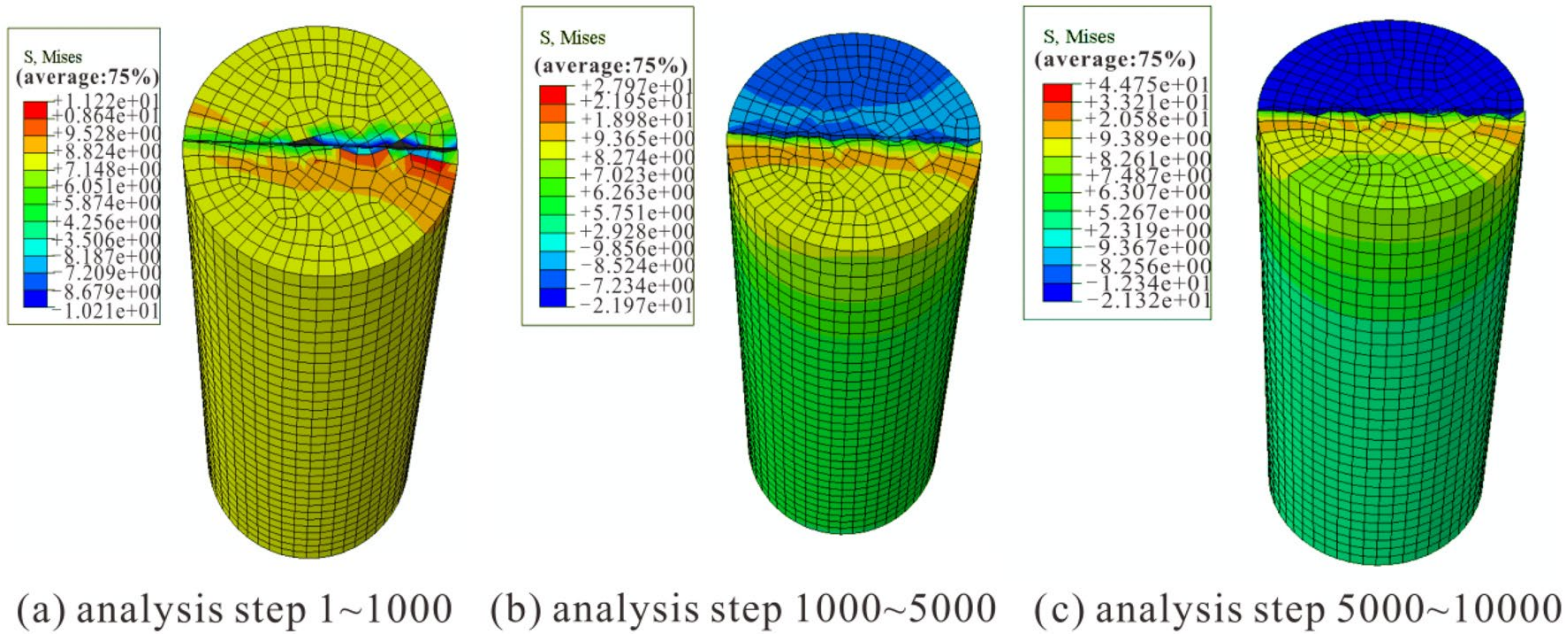
Numerical simulation stress cloud diagram after 1 dry–wet cycle.

**Figure 9:**
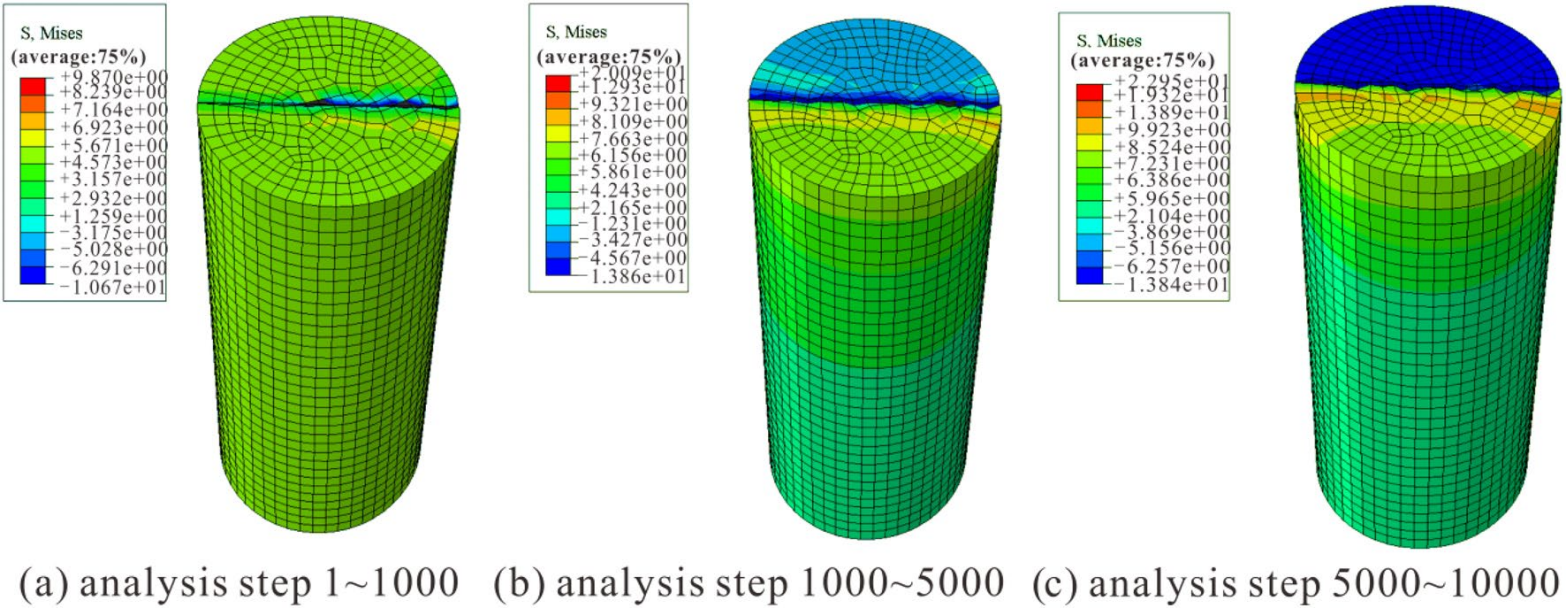
Numerical simulation stress cloud diagram after 10 dry–wet cycles.

The Mises stress cloud diagrams for uniaxial compression simulation of rock samples at various amounts of dry–wet cycles are shown in Figs. 7, 8 and 9. Comparing the stress results in the stress cloud plots, it can be clearly seen that the dry and wet cycles lead to a decrease in the peak stress of the sandstone, which is consistent with the experimental results and the changing trend of the numerical simulation results by Kang et al.^40^ and Wang et al.^41^. This further illustrates the feasibility of the numerical simulation.

The stress evolution process of the three models was analyzed, and it was found that all three models can be divided into three distinct loading stages with significant changes: when the number of loading steps is 1–1000, stress concentration first appears in the lower part of the middle of the model, followed by stress concentration in the lower right boundary of the model; when the number of loading steps is 1000–5000, the stress rises with the increasing analytical steps, and the stress concentration area gradually spreads to the surroundings of the model; when the number of loading steps is 5000–10,000, the area of stress concentration continues to spread until it penetrates the top and bottom of the model, and with further loading, the model sample is completely destroyed.

Y-1, Y-2, and Y-5 simulate 0, 1, and 10 dry–wet cycles, respectively, resulting in different stress changes in the three stages of the failure process (Table 3). The maximum stress appears in the 5000–10,000 steps of Y-1, with a maximum stress of 59.21 MPa, and the minimum stress appears in the 1–1000 steps of Y-3, with a minimum stress of 9.87 MPa. It is evident that the rock sample's mechanical stress diminishes with more dry–wet cycles, and the experimental and simulated results are in good agreement.

**Table 3:** Numerical simulation stress calculation results.

Various amounts of dry–wet cycles	Sample number	Analyzing steps	Maximum stress/MPa
0	Y-1	1–1000	14.77
		1000–5000	37.20
		5000–10,000	59.21
1	Y-2	1–1000	11.22
		1000–5000	27.97
		5000–10,000	41.05
10	Y-5	1–1000	9.87
		1000–5000	20.09
		5000–10,000	22.95

## Discussion

### Comparison of stress–strain curves and peak strength

**Figure 10:**
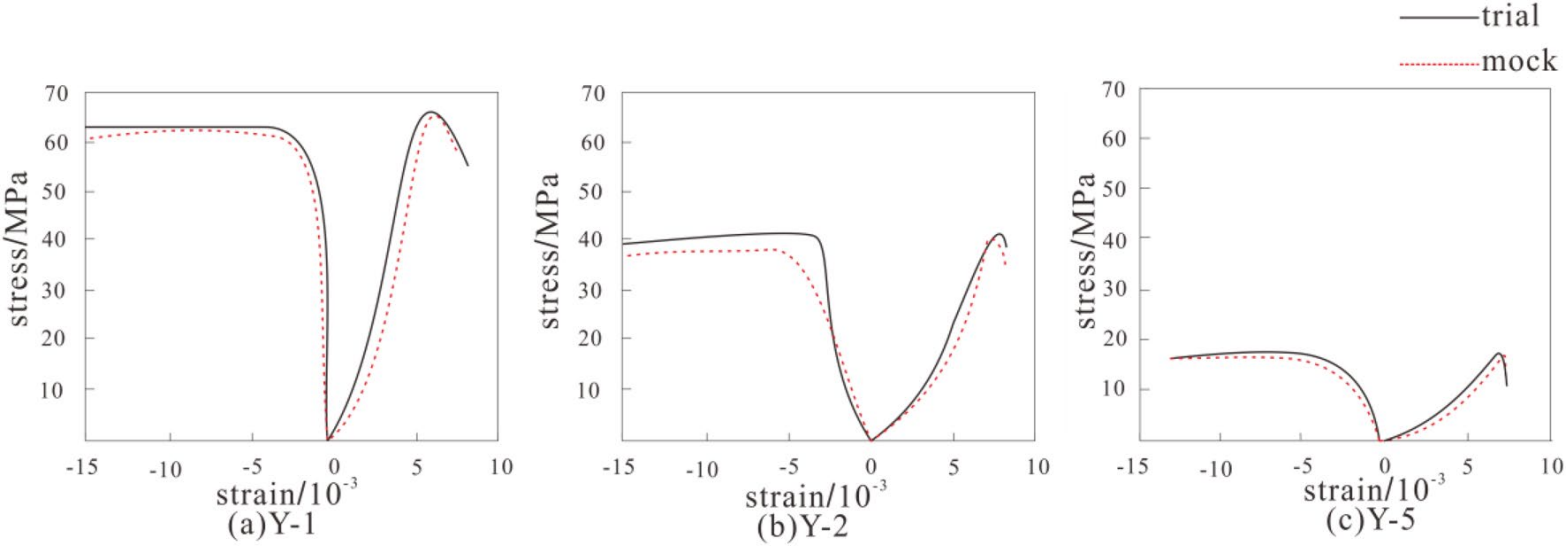
comparison of the stress–strain curves from simulation and experimentation: (**a**) Y-1, (**b**) Y-2, (**c**) Y-5.

The data obtained from numerical simulation and experiments of single axis compression various amounts of dry–wet cycles were compared and examined to produce a graph comparing their stress–strain curves (as depicted in Fig. 10), and the comparison table of peak stress is given in Table 4.

**Table 4:** Comparison of simulated and experimental values for dry–wet cycle simulation.

Various amounts of dry–wet cycles	Sample number	Peak stress simulation value/MPa	Peak stress test value/MPa	Error/%
0	Y-1	59.21	60.53	2.2
1	Y-2	41.05	42.35	3.1
10	Y-5	22.95	23.54	2.5

Examination of Fig. 10 reveals that the simulated and experimental curves exhibit similar patterns, characterized by five distinct stages: initial compaction, linear elastic deformation, stable crack expansion, pre-peak plastic hardening, and failure. All three samples failed at the maximum axial strain, and the corresponding strain values for the experiments and simulations are consistent, namely 0.008, 0.009, and 0.007, respectively. This indicates the consistency between the simulation and experiments, demonstrating the feasibility and necessity of the simulation.

Analysis of Table 4 shows that the peak stress obtained from ABAQUS simulation is close to the peak stress obtained from uniaxial compression tests, with a maximum error of only 3.1%. Through the literature review, it is found that the simulation comparison results are in good agreement with the previous results^42,43^, which further shows the consistency between the simulation curves and the test curves, and proves the feasibility and applicability of the simulation, as well as the applicability and correctness of the ABAQUS software in the simulation of the dry and wet cyclic damage process of rocks.

### Effect of dry and wet cycling on crack changes

**Figure 11:**
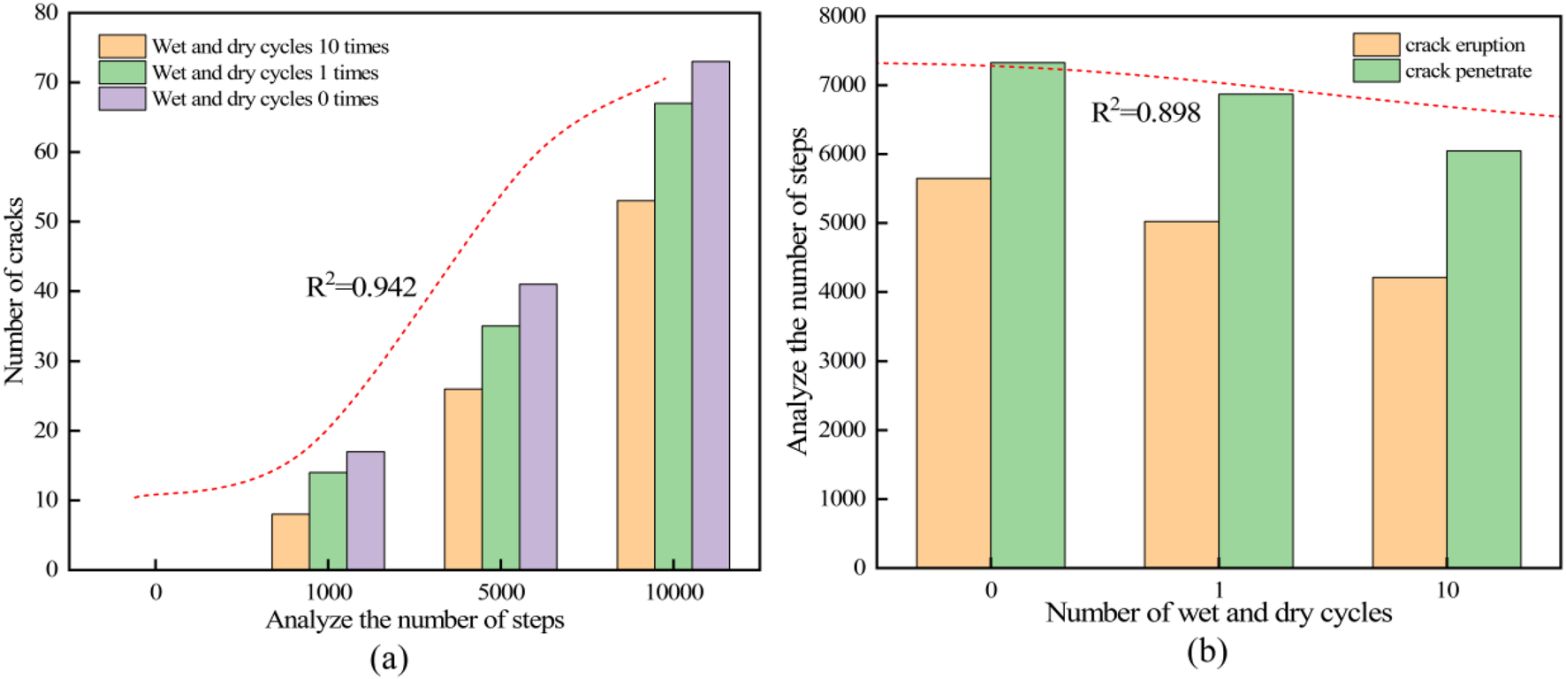
Crack variation plot in simulation: (**a**) changes in the number of cracks at different analysis steps for specimens with different quantities of dry–wet cycles. (**b**) Analysis steps of crack initiation and penetration for specimens with different quantities of dry–wet cycles.

The analysis of the complete process of the three models described above reveals the relationship between the number of cracks at different analysis steps (Fig. 11a) and the crack initiation and penetration analysis steps for specimens with different number of cycles (Fig. 11b). The analysis of Fig. 11a shows that the number of cracks increases with the higher number of wet and dry cycles, which is consistent with the findings of Liu et al.^44^ and Liu et al.^45^ in rock studies. This may be due to the effects of factors such as physical and chemical interactions between the water and the rock, which result in a one-time increase in the penetration of the water molecules and a decrease in the load-bearing capacity of the sandstone specimens. The originally homogeneous and dense sandstone specimens exhibit increasing numbers of loose particles, leading to increased fracture formation.

Analysis of Fig. 11b. reveals that for the sandstone specimen without dry–wet cycle treatment, crack initiation occurred at 5847 steps, and crack penetration occurred at 7325 steps. For the sandstone specimen subjected to 1 dry–wet cycle, crack initiation occurred at 5023 steps, and crack penetration occurred at 6269 steps. For the sandstone specimen subjected to 10 dry–wet cycles, crack initiation occurred at 4816 steps, and crack penetration occurred at 5920 steps. With more dry–wet cycles, the analysis steps corresponding to crack initiation and penetration for the sandstone specimen are advanced, and the quantity of analysis steps from crack initiation to penetration is reduced, indirectly indicating the continuous accumulation of internal damage in the sandstone specimen with more dry–wet cycles. The findings were in agreement with the results of Liu et al.^45^ and Chen et al.^46^ on sandstone research. A linear regression of the number of analysis steps and the number of cracks in sandstone with different numbers of wet and dry cycles and the number of cracks produced and the crack penetration in sandstone with different numbers of wet and dry cycles showed that the fitting coefficients were 0.942 and 0.898, respectively, which further showed that with the increase of the number of cycles, the compressive strength of sandstone gradually decreased, the extension of the cracks intensified, and sandstone was more likely to tend to damage.

Therefore, investigating the affect of dry–wet cycles on rocks could enhance our understanding of the evolution process and property changes of rocks, providing scientific basis and technical support for engineering construction and geological environmental protection.

## Conclusion

1. There are few studies using numerical simulation methods to study the crack extension of rocks under various dry and wet cycling conditions. In this paper, numerical simulation of dry and wet cycling tests and crack evolution of sandstone in Yunyang County, Three Gorges Reservoir Area, opens a new way to understand the evolution law of cracks under different environmental conditions, thus providing an important support for an in-depth understanding of the crack extension mechanism.
2. The number of dry and wet cycles does not change the deformation and fracture stages of sandstone, and uniaxial compression damage mainly includes initial compaction, linear elastic deformation, stable crack extension, pre-peak plastic hardening and destructive deformation. These stages are interrelated and constitute a complete damage process.
3. The uniaxial compressive strength and coefficient of recovery of sandstone decreased with the increase of the number of wet and dry cycles, on the contrary, Poisson's ratio showed a different pattern. It is noteworthy that the effect of the number of wet and dry cycles decreases with the increase in the number of cycles. The correlation coefficients obtained from fitting the three parameters with the amount of wet and dry cycles are all greater than 0.93, further indicating that the amount of wet and dry cycles plays an important role in significantly affecting the mechanical property indexes of sandstone.
4. Under different wet and dry cycling amounts, the deformation and damage processes were demonstrated to be consistent at the maximum axial strain by ABAQUS simulation. Specifically, the experimental data and numerical simulation results were 0.008, 0.007 and 0.009, respectively, showing a high degree of consistency. And the maximum error between the peak stress obtained from experiment and simulation is only 3.1%, which further verifies the high degree of agreement between simulation and experiment, and also confirms the applicability and accuracy of ABAQUS software in simulating the process of dry and wet cycling of rocks.
5. The numerical simulation results show that with the increase of the number of wet and dry cycles, the number of cracks in the sandstone specimens shows a gradual increase trend, which presents an obvious cumulative effect. At the same time, the analytical steps involved in the process of crack generation and penetration are also advanced, and the analytical steps from crack formation to complete penetration are reduced accordingly, which implies that the crack extension process tends to develop more quickly and rapidly in the wet and dry cycling.
6. This paper provides a new method for the numerical simulation of the mechanical properties of sandstone in wet and dry cycles, but the subsequent improvements in the numerical simulation of cracks in wet and dry cycles can include the consideration of multi-physical field coupling, the introduction of nonlinear effects, in-depth study of the crack extension mechanism, the combination of experimental data to verify the simulation results, the conduct of sensitivity analyses, and the development of multiscale simulation methods, and other measures. Through these improvements, the generation and expansion process of sandstone cracks can be simulated more comprehensively and accurately, and the crack behavior laws can be comprehensively investigated from micro to macro levels to ensure the reliability and accuracy of the simulation results, to provide stronger theoretical support and guidance for the application of sandstone engineering, and to promote the reliability and efficiency of engineering practice. Limited studies have utilized numerical simulation methods to investigate the crack propagation in rocks under various dry-wet cycling conditions. This approach offers a more detailed insight into the evolution patterns of cracks under different environmental scenarios, thereby providing crucial support for a deeper understanding of the mechanisms governing crack propagation.

## Data Availability

The data supporting the results of this study are in this paper. Others are available from the corresponding author.
